# Hombre de 18 años con síndrome verrugoso tropical: ¿leishmaniasis o esporotricosis?

**DOI:** 10.7705/biomedica.5757

**Published:** 2021-06-15

**Authors:** Paola Macías, Juliana Ordóñez, Claudia M. Arenas, Gerzaín Rodríguez

**Affiliations:** 1 Hospital Universitario Centro Dermatológico Federico Lleras Acosta, Bogotá, D.C., Colombia Hospital Universitario Centro Dermatológico Federico Lleras Acosta BogotáD.C. Colombia; 2 Fundación Universitaria Sanitas, Bogotá, D.C., Colombia Fundación Universitaria Sanitas BogotáD.C. Colombia; 3 Pontificia Universidad Javeriana, Bogotá, D.C., Colombia Pontificia Universidad Javeriana Pontificia Universidad Javeriana BogotáD.C. Colombia; 4 Universidad de La Sabana, Chía, Colombia Universidad de la Sabana Universidad de La Sabana Chía Colombia

**Keywords:** leishmaniasis cutánea, esporotricosis, dermatomicosis, micosis, Leishmaniasis, cutaneous, sporotrichosis, dermatomycosis, mycosis

## Abstract

El síndrome verrugoso tropical comprende condiciones cutáneas infecciosas, crónicas y granulomatosas que cursan con placas, nódulos o úlceras verrugosas, de ahí su nombre. Este síndrome incluye la cromoblastomicosis, la esporotricosis, la paracoccidioidomicosis, la lobomicosis, la leishmaniasis y la tuberculosis cutánea verrugosa, todas ellas enfermedades de amplia distribución en áreas tropicales y subtropicales.

Sus diagnósticos pueden ser difíciles y confundirse entre sí, lo cual es más frecuente entre la esporotricosis y la leishmaniasis. Para distinguirlas se recurre a criterios clínicos y epidemiológicos, y a métodos diagnósticos como intradermorreacción, examen directo, biopsia, cultivo, inmunofluorescencia y PCR, algunos de los cuales no son de uso común. El diagnóstico preciso conduce al tratamiento adecuado.

Se presenta el caso de un hombre de 18 años con extensas placas verrugosas en una rodilla, inicialmente interpretadas como leishmaniasis verrugosa por la clínica, la epidemiología y la biopsia. Se le trató con Glucantime® durante 20 días, pero no presentó mejoría, por lo que se tomó una nueva biopsia que también se interpretó como leishmaniasis cutánea. La revisión de ambas biopsias evidenció inflamación con granulomas abscedados y presencia de cuerpos asteroides esporotricósicos, que condujeron al diagnóstico de esporotricosis, el cual se confirmó luego con el cultivo del hongo. Las lesiones remitieron con la administración de itraconazol.

La clínica y la epidemiología de la leishmaniasis y las de la esporotricosis pueden ser semejantes, por lo que la biopsia y los estudios de laboratorio son esenciales para establecer el diagnóstico. El cuerpo asteroide esporotricósico es patognomónico de esta entidad. Se revisaron los conceptos esenciales de estas condiciones y los criterios para diferenciarlas.

## Abstract (en)

The tropical verrucous syndrome includes infectious, chronic, and granulomatous skin conditions appearing with plaques, nodules, or ulcers with a warty surface which gives name to the syndrome. It includes forms of chromoblastomycosis, sporotrichosis, paracoccidioidomycosis, lobomycosis, leishmaniasis, and tuberculosis verrucosa cutis with ample distribution in tropical and subtropical areas. The diagnoses may be difficult and confused among them, especially between sporotrichosis and leishmaniasis.

Clinical, epidemiologic, intradermal reactions, direct smears, skin biopsies, cultures, immunofluorescence, and PCR are used to differentiate them, although several of these methods are not commonly used.

We present an 18-year-old man with extensive verrucous plaques in one knee interpreted by clinic, epidemiology, and biopsy as verrucous cutaneous leishmaniasis. He was treated with Glucantime® for 20 days without improvement. A new biopsy was made that was also interpreted as cutaneous leishmaniasis. The revision of both biopsies showed inflammation with abscessed granulomas and asteroid sporotrichotic bodies at the center of the granulomas that led to the diagnosis of sporotrichosis later confirmed by the fungus culture. The patient responded to the treatment with itraconazole. As clinical and epidemiological findings of leishmaniasis and sporotrichosis can be similar, skin biopsy and other paraclinical studies are necessary to establish a proper diagnosis. The asteroid sporotrichotic body is pathognomonic of this mycosis. We review here the essential concepts of leishmaniasis and sporotrichosis and the criteria to differentiate them.

## Caso clínico

**Figura 1:**
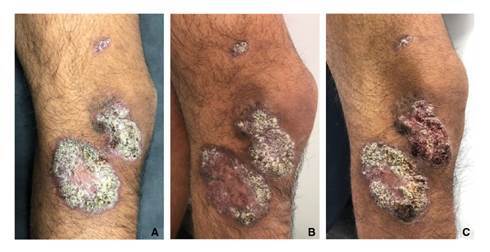
A. Placas verrugosas en la consulta inicial. B. Al terminar el tratamiento con Glucantime®, se observó una discreta disminución de la superficie verrugosa de la placa de la pierna. C. Un mes después, las lesiones eran semejantes a las iniciales.

Se trata de un hombre de 18 años de edad, estudiante y agricultor procedente del área rural de Chámeza, Casanare, Colombia. Hacía siete meses que presentaba placas verrugosas en la rodilla derecha, tenía antecedentes de picaduras de insectos y no había sufrido traumas. Tenía tres placas verrugosas, dos voluminosas, de bordes regulares y bien definidos en la cara externa de la rodilla y en el tercio superior de la pierna derecha, de 5 x 3 y 7 x 5 cm; la otra placa, en la cara antero-lateral del muslo, medía 13 x 7 mm (figura 1A).

Se sospechó leishmaniasis cutánea en el examen clínico, ya que es frecuente en la región de procedencia del paciente y por los antecedentes de picadura de insectos. Los dos frotis directos que se le tomaron fueron negativos para *Leishmania* spp. y se remitieron al Centro Dermatológico Federico Lleras para estudio. Allí se sugirieron los diagnósticos de leishmaniasis cutánea y, en segundo lugar, de esporotricosis.

Dados los antecedentes de frotis directos negativos, se tomó una biopsia de una de las lesiones, la cual mostró hiperplasia pseudocarcinomatosa moderada con capa córnea gruesa y paraqueratósica, pústulas intracórneas y dermis con infiltración difusa, granulomas de histiocitos epitelioides, algunas células gigantes multinucleadas y linfocitos, además de numerosas células plasmáticas y algunos cuerpos de Russell. Había conglomerados de neutrófilos en el epitelio y en el centro de algunos granulomas, y una cicatriz hipertrófica en la dermis profunda. Las coloraciones de Ziehl-Neelsen, PAS, Giemsa y Gomory no demostraron microorganismos. El informe sugirió leishmaniasis cutánea y resaltó la dificultad de demostrar los amastigotes en las formas crónicas de la enfermedad. Se diagnosticó leishmaniasis cutánea verrugosa y el paciente se trató con antimoniato de meglumina (Glucantime®) durante 20 días (20 mg/kg/día) después de confirmar que no hubiera alteraciones en el hemograma, los niveles de amilasa, el electrocardiograma, y las funciones renal y hepática.

Al finalizar el tratamiento, no se observó curación clínica (1): no disminuyó la induración de la base y no disminuyó el tamaño de la lesión en más del 50% del inicial, al contrario, las lesiones aumentaron de tamaño; solo una de ellas tenía centro cicatricial, pero persistían los cambios verrugosos en los bordes (figura 1).

Se tomó una nueva biopsia y se solicitó cultivo para hongos. En esta ocasión se informó hiperplasia pseudocarcinomatosa, abscesos córneos, y dermis con inflamación granulomatosa y algunos abscesos, abundantes células gigantes y áreas cicatriciales. Las estructuras puntiformes en el centro de los abscesos se interpretaron como muy sugestivas de *Leishmania* spp. y se diagnosticó leishmaniasis cutánea.

**Figura 2:**
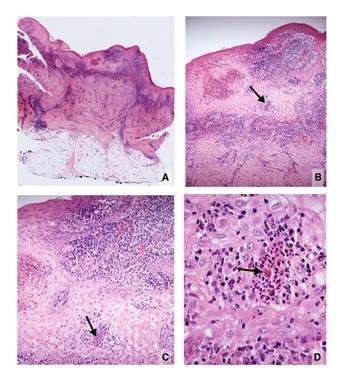
A. Imagen panorámica. Hiperplasia epidérmica e inflamación dérmica difusa con cicatriz profunda. Hipodermis normal. B. Hiperplasia epidérmica, abscesos intraepidérmicos, inflamación dérmica difusa y cicatriz. El absceso intraepidérmico (flecha), se amplía en C y D. C. Hiperplasia epidérmica y el mismo absceso diminuto intraepidérmico (flecha), con estructura eosinofílica central. D. Esta corresponde a una levadura central rodeada por espículas eosinófilas. Es un típico cuerpo asteroide esporotricósico. Hematoxilina y eosina, A: 2,5X; B: 10X; C: 20X; D: 80X.

**Figura 3:**
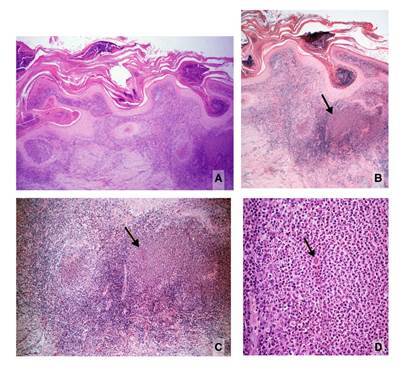
A. Imagen panorámica de la segunda biopsia. Hiperplasia epidérmica, abscesos córneos, senos de drenaje e inflamación dérmica difusa con granulomas centrados por abscesos, uno de ellos voluminoso (derecha). En el centro de la dermis hay cicatriz y un conglomerado de células gigantes. B. Área derecha de la figura anterior. Abscesos córneos y granulomas dérmicos abscedados. La flecha indica un punto eosinófilo en el absceso. C. Mayor aumento de B. Se ven granulomas abscedados y un cuerpo asteroide en el absceso de la derecha correspondiente al punto señalado en la figura anterior. Hay cicatriz dérmica profunda. D. Mayor aumento de C. Cuerpo asteroide esporotricósico dentro del absceso. Hay abundantes plasmocitos periféricos y cuerpos de Russell. Hematoxilina y eosina, A: 4X; B: 4X; C: 10X; D: 40X

Se solicitó a uno de los autores revisar las biopsias. En la inicial, se observó hiperplasia pseudocarcinomatosa moderada, abscesos córneos y paraqueratosis, así como dermis con inflamación difusa granulomatosa y la presencia ocasional de granulomas mixtos, es decir, con abscesos centrales. Los plasmocitos, linfocitos e histiocitos vacuolados eran abundantes y dispersos. Luego de cortes seriados, se observó en el centro de un pequeño absceso intraepidérmico un cuerpo asteroide, por lo cual se estableció plenamente el diagnóstico de esporotricosis (figura 2). La nueva biopsia evidenció cambios semejantes, con granulomas mixtos más aparentes y la presencia de un cuerpo asteroide esporotricósico en uno de ellos (figura 3). En el cultivo creció *Sporothrix* spp. El paciente se trató con 200 mg/día de itraconazol, con una mejoría significativa a los cuatro meses de tratamiento.

## Discusión

Este caso demuestra la dificultad para hacer el diagnóstico diferencial clínico e histológico entre la leishmaniasis cutánea y la esporotricosis, ambas incluidas en el síndrome verrugoso tropical. El paciente procedía de un área geográfica tropical, selvática, con alta prevalencia de leishmaniasis, en la que también es frecuente la esporotricosis (2-5). La clínica era sugestiva de leishmaniasis cutánea verrugosa por la presencia de placas hiperqueratósicas, vegetantes, cubiertas por escamo-costras gruesas y adherentes (1,2,6). El examen directo suele demostrar amastigotes en el 85 a 90% de los casos incipientes, pero con frecuencia es negativo en los casos crónicos y este resultado no descarta la posibilidad de leishmaniasis (1). En el presente caso, no se practicó PCR ni prueba de Montenegro (leishmanina), cuyos resultados positivos podrían deberse a la presencia de leishmaniasis activa o a la infección asintomática, pues el paciente habitaba en un área endémica para esta enfermedad (1). Por otra parte, la prueba de Montenegro ha sido positiva en casos de esporotricosis, lo cual se ha atribuido a componentes del reactivo, a enfermedad concomitante o a reacción cruzada (7). Un resultado negativo hubiera sido un hallazgo determinante en contra del diagnóstico de leishmaniasis. En este caso tampoco se practicó la prueba de reacción intradérmica de esporotricina.

De especial interés es la interpretación de la biopsia que tanto en la leishmaniasis verrugosa como en la esporotricosis demuestra hiperplasia pseudocarcinomatosa e inflamación dérmica granulomatosa rica en plasmocitos con cuerpos de Russell (2,8). En el 72% de los casos con menos de dos meses de evolución, se observan amastigotes en la biopsia y, en casos crónicos como el de este paciente, son muy difíciles de detectar (1,2). El patrón histológico de la leishmaniasis no incluye granulomas centrados por abscesos, que es la característica esencial para diferenciarla de la esporotricosis, en la cual estos se hallan presentes (1,2,6,8). Los abscesos intraepidérmicos dentro de los granulomas que se eliminan por esta vía, se presentan en la esporotricosis y no en la leishmaniasis; incluso, la presencia de pequeños abscesos aislados o en el centro de los granulomas dérmicos debe suscitar la sospecha de esporotricosis, como ocurrió en este caso. En las leishmaniasis es posible ver abscesos intraepidérmicos, especialmente si hay infección secundaria. Esto sucedió en seis casos de leishmaniasis cutánea en Marruecos, en los cuales se demostraron granulomas abscedados con compromiso folicular y presencia de amastigotes en los abscesos, hallazgo este excepcional (9). Además de los granulomas abscedados en la esporotricosis, la abundancia y la prominencia de células gigantes (segunda biopsia) y la cicatriz prominente (primera biopsia), son hallazgos típicos de la esporotricosis, no de la leishmaniasis cutánea (1,2).

En la esporotricosis es raro encontrar las “levaduras en cigarro” del hongo, fagocitadas o libres, incluso con coloraciones especiales (2,5,6), en cambio, se demuestra el cuerpo asteroide en los abscesos centrales de los granulomas dérmicos o en los abscesos intraepidérmicos hasta en el 70% de los casos (1,10,11). En el presente caso, su presencia estableció el diagnóstico de esporotricosis, confirmado luego con el cultivo positivo para *Sporothrix* spp. Es posible que en algunos de estos abscesos se observen solo las espículas eosinófilas, pero que, en el siguiente corte, aparezca la levadura, a veces en gemación, rodeada por las espículas (figuras 2, B y C). Si ello no ocurre, la sola presencia de las espículas es evidencia suficiente para hacer el diagnóstico histológico de esporotricosis (11,12). 

Los cuerpos asteroides esporotricósicos consisten en la levadura rodeada de espículas eosinófilas y corresponden a un típico fenómeno de Splendore-Hoeppli, que se interpreta como la precipitación de anticuerpos alrededor de un microorganismo o de un cuerpo extraño (10,12,13). También, podrían contener enzimas provenientes de la degranulación de los neutrófilos, una posibilidad que no se ha demostrado. Durante años se los confundió con los cuerpos asteroides citoplasmáticos de las células gigantes, resultantes de la fusión de macrófagos para formarlas (14).

Alfonso Splendore, médico italiano que trabajó varias décadas en São Paulo, Brasil, los describió en 1908 en una mujer italiana con esporotricosis de la cara (15). Hoeppli describió el mismo fenómeno en 1932, en China, alrededor de huevos de esquistosoma (10,12,13).

La frecuencia con la cual se demuestra el cuerpo asteroide esporotricósico es muy variable y posiblemente dependa de las especies del hongo (*S. schenkii sensu strictu*, *S. brasiliensis*, *S. lurie*, *S. globosa*, *S. mexicana*), de la reacción inmunitaria del huésped y del interés del patólogo en demostrarlo, lo que implica hacer, por lo menos, tres láminas con cortes seriados y observar con cuidado cada absceso o granuloma supurado (10,11). Por ejemplo, en la epidemia de esporotricosis de Río de Janeiro, producida por *S. brasiliensis*, de la cual se han registrado 4.669 casos en humanos y 4.703 en gatos (16,17), el cuerpo asteroide no se evidenció pero el hongo fue muy abundante en estos tejidos, sobre todo en los gatos. Por otra parte, en Uruguay (*S. schenkii sensu strictu*), el cuerpo asteroide se demostró hasta en el 94% de los casos en el examen directo en fresco (18), un procedimiento que se debería enfatizar en el estudio de esta micosis, pues se acepta como verdad que el examen directo para el estudio de la esporotricosis es inútil porque siempre es negativo (2,10,19). Aquí se le considera patognomónico porque su tamaño, localización y morfología son únicos y característicos (1,2,10-12,14,20), aunque algunos lo consideran inespecífico, concepto que no compartimos (19). Lo que es general y común es el fenómeno de Splendore-Hoeppli, que representa el depósito radiado de material eosinófilo, probablemente de anticuerpos, alrededor de bacterias, hongos o parásitos, como en la actinomicosis, la nocardiosis, la botriomicosis, la candidiasis, la conidiobolomicosis y la esquistosomiasis (10,12,13). La morfología que origina este fenómeno y la causa que lo desencadena son diferentes y, en la esporotricosis, ofrece una imagen típica y patognomónica.

Es posible que la biopsia no sea concluyente en cuanto a si se trata de esporotricosis o de leishmaniasis. En estos casos, el informe debe ser preciso. El médico tratante tiene la opción del cultivo de ambos gérmenes, pues los dos crecen en el medio de cultivo de Séneca, lo que constituye una ayuda excepcional (Zulma Alvarado, Laboratorio de Micología, Centro Dermatológico Federico Lleras, Bogotá, comunicación personal). Si se recurre al tratamiento de prueba, como en este caso, la falta de un resultado satisfactorio es una indicación de que no se trata de la enfermedad que se ha considerado. No obstante, el Glucantime® se ha usado para tratar la esporotricosis y el itraconazol para la leishmaniasis cutánea con efectos aceptables (7). Por otra parte, la infección mixta de leishmaniasis y esporotricosis en las mismas lesiones se ha demostrada en tres pacientes colombianos (21). La elucidación diagnóstica lleva al tratamiento adecuado, como sucedió en el presente caso.

En Colombia se diagnostican anualmente entre 10.000 y 12.000 casos nuevos de leishmaniasis, el 97,5% de los cuales corresponde a leishmaniasis cutánea (1). Se observan numerosas formas clínicas, entre las cuales la apariencia verrugosa no es rara (1). Debe contemplarse el diagnóstico diferencial con todos los componentes del síndrome verrugoso tropical, especialmente con la esporotricosis, la micosis profunda más frecuente en nuestro país y en el mundo, asociada con traumas ocasionados por material vegetal, contactos con animales e, inclusive, con la picadura de insectos (3-5). Esta condición se incluye entre las micosis de implantación y afecta a pacientes de cualquier edad con lesiones predominantemente en los brazos y en las piernas, lo que se presenta igualmente en la leishmaniasis, con la cual comparte, además, la forma linfangítica (1).

## Conclusiones

El síndrome verrugoso tropical agrupa enfermedades con características clínicas e histológicas similares, por lo que es importante hacer una adecuada correlación clínico-patológica que permita llegar a un diagnóstico certero y brindar un tratamiento adecuado. Las condiciones patológicas de este síndrome que más se confunden entre sí son la leishmaniasis y la esporotricosis, que ocurren en los mismos hábitats. El examen directo, las intradermorreacciones, el cultivo, la biopsia, la biología molecular y la prueba terapéutica, son procedimientos decisivos para establecer el diagnóstico preciso, pero pocos están disponibles. Por eso, es necesario usarlos con eficiencia, pues los tratamientos son costosos y prolongados, y en el caso de la leishmaniasis, tienen riesgos importantes. En la esporotricosis, se debe buscar el cuerpo asteroide o sus espículas, que tienen valor diagnóstico patognomónico.
